# Spontaneous hemoperitoneum in a 29‐week pregnancy with a history of endometriosis: A case report and review of the literature

**DOI:** 10.1002/ijgo.16006

**Published:** 2024-11-11

**Authors:** Shamsi Mehdiyev, Fatma Basak Tanoglu, Esma Demir Altuncu, Engin Oral

**Affiliations:** ^1^ Department of Obstetrics and Gynecology Bezmialem Vakif University School of Medicine Istanbul Turkey; ^2^ Department of Obstetrics and Gynecology Biruni University School of Medicine Istanbul Turkey

**Keywords:** assisted reproductive technology, endometriosis, hemoperitoneum, maternal morbidity, perinatal mortality, pregnancy, surgery

## Abstract

Spontaneous hemoperitoneum in pregnancy (SHIP) is defined as sudden, nontraumatic intraperitoneal bleeding that occurs during pregnancy or up to 42 days postpartum. The incidence ranges between 4 and 4.9 per 100 000 births. Although seen rarely, it is associated with perinatal morbidity and mortality due to maternal hemodynamic instability. Endometriosis was shown to be present in 71% of SHIP cases. A 30‐year‐old primigravid woman with a spontaneous conception, at 29 weeks of gestation, presented to our obstetrics and gynecology emergency department with complaints of abdominal and back pain. In terms of her medical history, a laparoscopic cystectomy was performed in August 2022 due to a 90 mm × 50 mm endometrioma in the right ovary. However, deep endometriosis and adenomyosis were not observed. After decelerations appeared on the non‐stress test, the repeat hemoglobin values dropped to 7.2 g/dL, with blood pressure at 70/50 mm Hg and a pulse rate of 95/min. The decision was made for laparotomy and emergency delivery of the baby. It is crucial to consider SHIP, especially in pregnant patients with a history of endometriosis surgery. Managing such high‐risk cases in specialized centers and easily identifying predisposing factors for SHIP can lead to improved outcomes, despite its rarity and poor prognosis.

## Synopsis

Spontaneous hemoperitoneum should be kept in mind as an acute event associated with maternal–fetal mortality and morbidity among endometriosis‐associated complications in pregnancy.

## 1 INTRODUCTION

Endometriosis is a common gynecological disease that affects at least 10% of women in reproductive age.^1^ Although it is known as one of the causes of infertility, it is also associated with negative pregnancy outcomes. Miscarriage, preterm birth, placenta‐associated complications (pre‐eclampsia, abruptio placenta, and placenta previa), hemorrhage, acute abdomen (spontaneous uro‐hemoperitoneum, uterine rupture, and bowel perforation), fetal dystocia, stillbirth, and cesarean delivery are among these negative outcomes .^2^ Endometriosis was shown to be present in 71% of spontaneous hemoperitoneum in pregnancy (SHIP) cases.^3^ SHIP is defined as sudden, nontraumatic intraperitoneal bleeding that occurs during pregnancy or up to 42 days postpartum.^4^ The incidence ranges between 4 and 4.9 per 100 000 births.^5^ Although seen rarely, it is associated with perinatal morbidity and mortality due to maternal hemodynamic instability. Pregnancies in women older than 35, previous history of abdominal surgery, use of assisted reproductive technology (ART) for conception, multiple pregnancy, and endometriosis are some of the known risk factors for SHIP.^6^, ^7^ SHIP is most commonly encountered during the second half of pregnancy and necessitates emergency surgical intervention. This often results in high neonatal mortality rates secondary to preterm birth. Due to its nonspecific clinical presentation, including symptoms such as abdominal pain, signs of hypovolemic shock, and fetal distress, the final diagnosis is typically established intraoperatively. Despite its rare occurrence, early diagnosis and appropriate management are of utmost significance, given its potential to be a life‐threatening condition.

In this study, we present the case of a 30‐year‐old 29‐week pregnant patient with a history of endometriosis surgery who was admitted to our emergency department with SHIP.

## 2 CASE REPORT

A 30‐year‐old primigravid woman with a spontaneous conception, at 29 weeks of gestation, presented to our obstetrics and gynecology emergency department with complaints of abdominal and back pain. She had sought care at another center with similar complaints 1 week prior, leading to hospitalization. At that time, her cervical length was measured as 22 mm, and she was noted to have polyhydramnios. However, no complications were observed during follow‐up, and she was discharged 3 days later as her symptoms improved. In terms of her medical history, a laparoscopic cystectomy was performed in August 2022 due to a 90 mm × 50 mm endometrioma in the right ovary. However, deep endometriosis and adenomyosis were not observed. She achieved a spontaneous pregnancy 3 months after surgery.

At the time of arrival, the patient's vital signs were recorded as follows: pulse 77/min, blood pressure 80/40 mm Hg, temperature 36.2°C, and oxygen saturation of 99%. Obstetric ultrasound revealed a single fetus with a positive heartbeat, increased amniotic fluid volume, and an estimated fetal weight consistent with gestational age. The placenta was visualized as normal on the posterior uterine wall, and the cervical length measured 22 mm. No abundant free fluid or bleeding was observed, ruling out uterine rupture as a concern. The non‐stress test showed reactivity without any contractions. The patient's hemoglobin level at the time of arrival was 8.3 g/dL. Due to intensifying abdominal pain, a general surgery consultation was requested. Thirty minutes later, decelerations appeared on the non‐stress test, and repeat hemoglobin values dropped to 7.2 g/dL, with blood pressure at 70/50 mm Hg and a pulse rate of 95/min. Consequently, the decision was made for laparotomy and emergency delivery of the baby. The procedure was performed under general anesthesia, and entry was made into the abdominal cavity. Upon entering the abdomen, a significant amount of blood and coagulum was observed. Subsequently, the uterus was opened via a Kerr incision, and a male fetus weighing 1555 grams was delivered, with Apgar scores of 4 and 6. Umbilical cord blood gases revealed a pH of 6.8 and an anion gap of −21.4. The newborn was admitted to the Neonatal Intensive Care Unit (NICU). A wide exploration was conducted, involving the removal of a massive coagulum, which amounted to nearly 1000 cc of blood. An active bleeding site, displaying a decidual appearance, was identified on the left side of the uterus near the broad ligament. Bleeding was successfully controlled with sutures. Subsequently, due to observed bleeding in the upper abdomen near the spleen, a general surgeon was summoned to the operation. The current transverse incision was extended superiorly up to the umbilicus along the midline. As no active bleeding site was observed in this region, three drainage tubes were inserted, and the abdomen was closed. The patient received one unit of erythrocyte suspension intraoperatively and two units postoperatively. Additionally, only one unit of fresh frozen plasma (FFP) was infused during the postoperative period. The patient was discharged from the hospital on the fourth day. The newborn was discharged from the hospital on the 48th day with no complications.

The patient gave their free informed consent to the anonymous publication of this case. The Bezmialem University Hospital institutional review board (IRB). In committee decided that the publication of this case report did not require its approval.

## 3 DISCUSSION

Spontaneous hemoperitoneum in pregnancy, despite its rarity in medical practice, has been recognized for its poor maternal and fetal outcomes since the 18th century.^8^ It continues to pose challenges in terms of prompt diagnosis and management. For this case, we aimed to present the diagnosis and management of a 29‐week pregnant woman with a history of endometriosis surgery.

In the literature, there are 67 SHIP cases with endometriosis (Table 1). The mean age of the patients was 32.95 ± 4.39, and 68.18% of them were under 35 years of age. In addition, 77.61% of these patients were nulliparous and 14.93% were multiple pregnancies. Those who became pregnant with ART and those who had a history of previous abdominal surgery constituted 41.79% and 52.23% of the patients, respectively. The mean week when SHIP presented was 27.71 ± 7.86, while the week when pregnancy was terminated was calculated as 29.70 ± 7.90. In most cases, the bleeding site was the posterior surface of the uterus. The results together with the patients' initial symptoms, the median amount of bleeding and pregnancy outcomes are shown in Table 2.

**TABLE 1:** Summary of cases with spontaneous hemoperitoneum in pregnancy.

Case	Author, year	Maternal age	Parity	Site of bleeding	Blood loss (mL)	Gestational age at delivery (weeks)	Outcome
1	Izumi, 1989^9^	31	0	Branch of left uterine artery	2500	12	Miscarriage
2	Kawabata, 1991^10^	23	0	Vessels on the right anterior	1500	30	Live birth
3	Inoue, 1992^11^	37	0	Vessels on the upper anterior	3000	29	Live birth
4	Muzimoto, 1996^12^	28	0	Vessels on the fundus	4000	28	Neonatal death
5	Leung, 1998^13^	35	0	Surface of the uterus and the vesico‐uterine peritoneal fold	2000	33	Stillbirth
6	Ismail, 1999^14^	N/A	0	Back of the uterus distributed over the uterosacral ligaments	N/A	33	Live birth
7	Aziz, 2004^15^	30	0	Left broad ligament over the area of the left uterine artery	4000	20	Stillbirth
8	Katorza, 2007^16^	29	0	Posterior upper right surface of the uterus and the ovarian fossa	2000	28 (twin)	Live birth
9		31	1	Right adnexa and parametrium	3000	26	Stillbirth
10		32	0	Posterior surface of the uterus and right anterior surface of the uterus near the round ligament	small amount	29	Live birth
11	Wu, 2007^17^	31	1	Posterior serosal surface of the uterus	3000	32 (twin)	Live birth
12	Passos, 2008^18^	30	0	Left broad ligament	large clots	32 (twin)	Live birth
13		32	0	Right uterine vein on the posterior surface of the uterus and posterior surface of the left broad ligament	large clots	31	Live birth
14	Chiodo, 2008^19^	22	0	right uterine artery	2000	31	Stillbirth
15	Roche, 2008^20^	43	0	Right uterine vessels, surface of the uterus, right utero‐ovarian ligament	3000	33 (twin)	Stillbirth
16	Wada, 2009^21^	31	2	right uterine vein	2490	37	Live birth
17	Zhang, 2009^22^	38	0	Posterior wall of uterus varicosities	3100	29 (twin)	Stillbirth
18	Kim, 2010^23^	33	0	Posterior wall of uterus varicosities	2000	33 (twin)	Live birth
19		28	0	Posterior surface of the uterus	1000	25	Live birth
20		37	1	uterine fundus and pouch of Douglas adhesions	large clots	40	Live birth
21		29	0	pouch of Douglas adhesions	large clots	41	Live birth
22	Brouckaert, 2010^24^	33	0	Right ovary and broad ligament	14 600	17	miscarriage
23	Grunewald, 2010^25^	33	2	Right uterosacral ligament at 27 weeks	900	42	Live birth
24	Reif, 2011^26^	25	0	Left adnexa	1500	27 (twin)	Live birth
25	Tourette, 2011^27^	28	1	Posterior uterine wall, left uterine artery	massive	28	Live birth
26	Girard, 2012^28^	31	0	Left broad ligament, uterine artery	massive	41	neonatal death
27	De Vincenzo, 2013^29^	33	0	Posterior uterine surface, bowel, left uterine artery	2500	24	Stillbirth
28	Aggarwal, 2014^30^	31	0	Left fallopian tube	2200	22 (twin)	Stillbirth
29	Berlac, 2014^31^	34	0	Front, back and fundus of the uterus	1500	29	Live birth
30	Cozzolino, 2015^32^	33	1	Right ovary and from the right‐posterior surface of the uterus	1500	29	Live birth
31	Loh, 2015^33^	31	0	posterior wall of uterus, left fallopian tube	3500	22 (twin)	Stillbirth
32	Stochino, 2016^34^	26	0	Left broad ligament, left uterine artery	3000	16	miscarriage
33	Lier, 2017^35^	38	0	Posterior uterine varicosities	3000	39	Live birth
34		35	0	Left ovary, bowel	1100	28	Live birth
35		33	0	Right broad ligament varicosities	3500	32	Live birth
36		34	2	Uterosacral ligament	1000	36	Live birth
37		37	2	Uterosacral ligament	2000	37	Live birth
38		36	1	Right broad ligament, right uterine artery	2000	6	miscarriage
39		28	0	Back of the uterus, left and right broad ligament, bilateral hematoma	100	37	Live birth
40		37	0	Right broad ligament, right round ligament, left uterosacral ligament, bladder	1750	30	Live birth
41	Rafi, 2017^36^	41	0	Left uterosacral ligament	5000	32 (twin)	Live birth
42	Kollikonda, 2021^37^	32	0	Right posterior uterine wall	1500	40	Live birth
43	Huang, 2021^38^	35	0	Posterior uterine surface	1500	18	miscarrriage
44	Golfier, 2022^39^	29	0	Deep endometriosis	950	25	Live birth
45		32	1	anterior and posterior surfaces of the uterus	2600	13	miscarriage
46		31	0	A tear in the posterior face of the uterus at the utero‐ovarian level, from a ruptured ovarian adhesion	2300	17	miscarriage
47		36	1	Deep endometriosis	N/A	34	Live birth
48		30	0	Deep endometriosis	2000	22	Stillbirth
49		28	0	Deep endometriosis	2200	41	Live birth
50		34	0	Deep endometriosis	3400	28	Live birth
51		34	0	Deep endometriosis	400	32	Live birth
52		33	0	N/A	500	37	Live birth
53		40	2	N/A	N/A	39	Stillbirth
54		38	2	Deep endometriosis	900	38	Live birth
55	Pereira, 2022^40^	45	0	Left lower portion of the uterine Posterior surface	2000	35	Live birth
56	Gaia, 2022^41^	39	0	N/A	4000	15	miscarriage
57	Kato, 2023^6^	41	0	Posterior uterine wall	2020	28	Live birth
58	Hirata, 2023^42^	36	1	Left uterine vein and left ovary	massive	32	Live birth
59	Mamah, 2023^43^	30	0	Left adnexa	4700	33	Live birth
60	Bazzurini, 2023^44^	31–41	0	The right round ligament and pouch of Douglas infiltrating the rectal wall	300	37	Live birth
61			0	Pre‐vesical peritoneum	500	35	Live birth
62			0	The right parametrium and right ovary	5000	32	Live birth
63			0	Left posterior uterine wall, right uterosacral ligament	4500	28	Live birth
64			0	Posterior uterine wall and visceral peritoneum	4000	37	Live birth
65			0	Left paracervical tissue, ovary and salpinx	5000	39	Live birth
66	Ikagawa, 2024^45^	31	0	Anterior and posterior surfaces of the uterus, peritoneum in the vesicouterine excavation	6000	19	miscarriage
67	Our case, 2024	30	0	Left side of the uterus near the broad ligament	1000	29	Live birth

Abbreviation: N/A, not applicable.

**TABLE 2:** Demographic and clinic characteristics of patients with spontaneous hemoperitoneum in pregnancy.

Characteristics of patients (*N* = 67)	
Age (years) (*N* = 66)	32.95 ± 4.39 [22–45]
< 35	45 (68.18)
**≥** 35	21 (31.82)
Parity	
0	52 (77.61)
1	9 (13.44)
2	6 (8.95)
Pregnancy	
Single	57 (85.07)
Multiple	10 (14.93)
Use of ART for conception	28 (41.79)
Previous history of abdominal surgery	35 (52.23)
Symptoms	
Abdominal pain	51 (76.11)
Hypovolemic shock	27 (40.29)
Fetal distress	16 (23.88)
Gestational age at occurrence (weeks)	27.71 ± 7.86 [6–41]
Gestational age at delivery (weeks)	29.70 ± 7.90 [6–42]
Estimated blood loss (mL) (*N* = 56)	2110 [100–14 600]
Pregnancy outcomes (*N* = 77)	
Live birth	51 (66.23)
Stillbirth	15 (19.48)
Miscarriage	9 (11.69)
Neonatal death	2 (2.60)

*Note*: Data are expressed as mean ± standard deviation, median, minimum, maximum or number (%).

Abbreviation: ART, assisted reproductive technology.

In a prospective population‐based cohort study conducted by Mazzacco et al.^46^ in Italy, it was reported that at the time of hospital admission abdominal pain was present in 89.6% of SHIP patients, hypotensive symptoms in 27.6%, hypovolemic shock in 24.1%, and fetal distress in 37.9%. The differential diagnosis includes conditions involving extra‐pelvic structures such as traumatic splenic artery and vein bleeding, splenic and hepatic injury, and ovarian artery rupture. Additionally, cases involving uterine rupture and placental abruption, which present with abdominal pain and fetal distress, can also lead to massive bleeding. Challenges in early diagnosis arise from symptoms that are non‐specific and can mimic other conditions. A comprehensive review of current literature suggests that early assessment of these patients, manifesting hemodynamic instability or fetal bradycardia, warrants emergency laparotomy without delay for imaging methods. In instances where hemodynamic stability and fetal well‐being are confirmed through imaging modalities such as ultrasound, computed tomography, or magnetic resonance imaging, the application of minimally invasive techniques such as laparoscopy and artery embolization facilitates the continuation of pregnancy and might even prevent complications in newborns resulting from preterm births.^39^, ^47^, ^48^ Given the presence of symptoms such as back and abdominal pain, hypotensive symptoms, and later, the manifestation of fetal distress after initial follow‐up in our patient, a definitive diagnosis was established following explorative laparotomy. However, diagnostic imaging modalities could be used in cases lacking hemodynamic instability and reassuring fetal well‐being. Expectant management could then be considered as an alternative option, aiming to reduce urgent C‐section rates and mitigate negative neonatal outcomes.

Although ectopic pregnancy and miscarriages are known to be complications related to endometriosis during pregnancy, SHIP is an acute event associated with maternal and fetal mortality and morbidity that should be kept in mind with a prevalence of 0.4%.^49^ When Lier et al.,^50^ examined the intraoperative biopsies taken from the areas causing bleeding in SHIP cases, they found that the pathological diagnosis was endometriosis in 75% and deciduosis in 25%. Aziz et al.^15^ demonstrated medial irregularity, fragility, and widening of vessels lumen and even loss of vascular integrity of vessels in endometriotic lesions. To meet the increasing needs of the placenta and fetus, an increase in plasma volume occurs as pregnancy progresses, and this increase begins to become evident as early as 6–8 weeks and reaches its peak in the 32nd week of pregnancy.^51^ Bleeding arises both from increased intraluminal pressure caused by pregnancy and vascular erosions secondary to chronic inflammation prompted by endometriosis.^52^ The association of SHIP cases with third trimester can be related to the fact that the anti‐inflammatory effects of progesterone decrease as gestation advances to the third trimester.^44^ This process prompts inflammation and peritoneal hemorrhages. Similar to our case, a review of cases by Mazocca et al.^46^ reveals that 65% of SHIP cases had previously undergone abdominal surgery, which increases intrabdominal inflammation secondary to previous surgery, fragility of vessel walls, and increased peritoneal tension due to abdominal adhesions predisposed to bleeding.

The maternal mortality rate, which was 49% in the review conducted by Hodgkinson et al.,^53^ encompassing 75 cases in 1950, has significantly declined to 0%–0.4% due to developments in advanced life support methods, modernization in anesthesia, and improved surgical techniques. However, perinatal mortality rates remain persistently high, ranging from 31% to 36%. By successfully navigating diagnostic pathways, expectant management can be considered as an alternative to emergency C‐section. Additionally, the use of embolization and the availability and feasibility of laparoscopic approaches might contribute to reducing preterm birth rates and mitigating negative neonatal outcomes by allowing the continuation of pregnancy.

In conclusion, it is crucial to consider SHIP in pregnancy, especially in pregnant patients with a history of endometriosis surgery. Managing such high‐risk cases in specialized centers and easily identifying predisposing factors for SHIP can lead to improved outcomes, despite its rarity and poor prognosis.

## AUTHOR CONTRIBUTIONS

Shamsi Mehdiyev—Data acquisition, manuscript drafting. Fatma Basak Tanoglu—Data interpretation, manuscript drafting. Esma Demir Altuncu—Data acquisition. Engin Oral— Conception, final approval.

## FUNDING INFORMATION

No funding was received for preparing this manuscript.

## CONFLICT OF INTEREST STATEMENT

The authors declare they have nothing to disclose.

## Data Availability

Data sharing is not applicable to this article as no new data were created or analyzed in this study.
